# Use of Non-Pharmacological Approaches for Migraine Treatment: Results from the Migraine in Aotearoa New Zealand Survey

**DOI:** 10.3390/jcm14124023

**Published:** 2025-06-06

**Authors:** Fiona Imlach, Susan Garrett

**Affiliations:** 1 Department of Public Health, University of Otago Wellington, Wellington 6242, New Zealand; 2 Department of Primary Health Care and General Practice, University of Otago Wellington, Wellington 6242, New Zealand; sue.garrett@otago.ac.nz

**Keywords:** migraine disorders, migraine treatment, migraine prevention, supplements, complementary therapy, non-pharmacological treatment, integrative treatment, online survey, New Zealand

## Abstract

**Background/Objectives:** Migraine is a common neurological disease with a high disability burden. Despite this, many people with migraine do not take medication to prevent attacks, even when this is indicated. Many non-pharmacological approaches to migraine treatment exist, but little is known about how people with migraine use these options. **Methods:** The online Migraine in Aotearoa New Zealand (NZ) Survey collected responses from 530 people with migraine from August to October 2022. Questions included current and previous use of preventive medications, supplements (e.g., magnesium, riboflavin, coenzyme Q10) and complementary therapies (e.g., meditation, biofeedback, yoga, acupuncture). **Results:** Around half of the respondents were currently using a supplement, 58% were currently using complementary therapy and half were taking preventive medication. One in five were using all three approaches for migraine prevention. Of those not taking preventive medication, 44% were taking a supplement, and 53% were using complementary therapy. For commonly used non-pharmacological approaches, 20–30% of people had tried them in the past but stopped due to lack of efficacy or other reasons. A high proportion of people would like to try approaches that are not readily available or expensive in NZ (e.g., neurostimulation devices and biofeedback). **Conclusions:** The use of supplements and complementary therapies for migraine prevention is common, often in combination with medication. There are high levels of discontinuation due to ineffectiveness and cost prohibits use for many. Improved access to non-pharmacological therapies, particularly as an adjunct, has the potential to improve outcomes for people with migraine, but more and better migraine treatment options are also needed.

## 1. Introduction

Migraine is a common, complex neurological disease affecting over a billion people worldwide and ranked as the 4th highest cause of years lived with disability, but the 2nd highest cause for young adults aged 15–49 years, from the Global Burden of Disease study in 2021 [1]. In Aotearoa (New Zealand (NZ)), an estimated 753,000 people live with migraine [2], with significant impacts on work and quality of life [3].

Migraine is characterized by recurrent headache, typically moderate–severe, one-sided and pulsating in nature, often accompanied by nausea, photophobia or phonophobia, and preceded and followed by other symptoms such as fatigue, neck stiffness, mood changes and cravings [4]. Migraine can become chronic, defined by the International Headache Society as a headache occurring 15 days or more a month (with migraine features on at least eight days) for at least three months [4], which occurs in 7–9% of people with migraine and causes high levels of disability [5]. When migraine attacks are frequent and/or severe, preventive medication is recommended to reduce the frequency and disability from attacks [6]. However, many people who have been diagnosed with migraine, including chronic migraine, do not take medication to prevent attacks even when this is indicated, often due to low tolerability and low efficacy of commonly prescribed preventives [7,8,9,10]. In NZ, a survey of people with migraine in 2022 found that only 57% who were eligible for preventive medication under strict criteria of frequency and severity of attacks were currently taking it [11]. Only 28% of those with headache on 15 days or more a month were taking a preventive, and this group had previously tried an average of four preventive medications but had stopped these primarily because of side effects or low efficacy [11]. Clinically, an effective preventive medication is defined as one that reduces attack frequency by 50%, but this may still leave people with chronic migraine with a significant headache burden.

Non-pharmacological options can be useful as adjunctive approaches to migraine prevention and also stand-alone measures when medication is not tolerated, contraindicated or not wanted by the patient, for example, during pregnancy. These options are often backed by lower-quality evidence than medications; however, most are considered safe with few side effects [12], with exceptions mentioned below.

Supplements that have at least some evidence for effectiveness in migraine prevention include magnesium, riboflavin (vitamin B2), coenzyme Q10, feverfew and butterbur [13,14]. Of note, there have been concerns about potential liver toxicity with the use of butterbur, which has led some countries to withdraw this product from the market, and feverfew is not recommended in pregnancy [14].

Complementary or integrative therapies with some evidence for effectiveness in migraine prevention include acupuncture, massage, meditation/mindfulness, cognitive behavioral therapy, yoga and Tai Chi, biofeedback, relaxation training, physical therapy and hypnotherapy [12,13,15,16,17,18]. The evidence for some other therapies is mixed or weak and cautions are made regarding spinal manipulative therapy due to the rare but serious and potentially life-threatening complication of cervical artery dissection [14,17]. Neuromodulation devices represent a new wave of non-pharmacological therapy and act to reduce pain through stimulation of the nervous system by the application of an electric current or magnetic field. There are currently four devices that have been approved for use in migraine [6,19] but only one (the supra-orbital transcutaneous electric nerve stimulator) has some availability in NZ, but this is limited and comes at a cost.

Although non-pharmacological therapies are widely recommended for migraine management, little is known about how these are used among people with migraine, with most studies being conducted in headache centers [20,21,22]. In a general population-based survey from the US, use of complementary therapies was found to be higher among those with migraine or severe headache (50%) than those without (34%), but only a small minority of people with migraine (5%) reported using these therapies as migraine treatment [23]. By contrast, from a survey of people with migraine in Germany, almost half had used non-pharmacological preventive approaches, with higher rates of use among those with chronic migraine [8]. A systematic review of non-pharmacological strategies used to manage migraine in the community found only 13 studies, but these were focused on pain management during attacks, not prevention [24].

No previous research has investigated the use of non-pharmacological approaches for migraine prevention in NZ. Through an online survey of people in NZ with migraine, we aimed to:

- Assess the use of supplements and complementary therapies (overall and for specific therapies) for migraine prevention, compared to prescribed medication.
- Compare the use of supplements and complementary therapies in those with a high frequency of monthly headache days (15 days or more a month) to those with headache on 14 days or fewer a month.
- Document reasons for discontinuing non-pharmacological treatments and assess potential unmet need (those who had not previously used these treatments but would like to).

## 2. Materials and Methods

### 2.1. Study Population and Recruitment

The study population included people with migraine living in NZ at the time of the survey. Survey recruitment was conducted through multiple channels, including social media and networks of Migraine Foundation Aotearoa New Zealand (MFANZ), the patient-facing education website Health Navigator (now Healthify), the Neurological Foundation of NZ and the NZ Pain Society. Additional promotion was achieved through a dedicated link on MFANZ’s website and media articles. The survey was available online from 22 August to 7 September 2022, delivered via SurveyMonkey (www.surveymonkey.com, San Mateo, CA, USA).

From an initial 579 responses, 4 duplicates and 33 respondents who had abandoned the survey early (completing 6% or less of survey items) were removed. The final sample of 530 respondents included only those with a positive result on the Migraine Identification test (ID-Migraine^TM^) [25] (n = 513) or those who reported having received a migraine diagnosis from a health professional (n = 17). The ID-Migraine^TM^ consists of three questions that screen for symptoms of migraine and is considered positive if respondents answer ‘yes’ to at least two of the questions, with a sensitivity of 84% and specificity of 76% [26].

### 2.2. Survey Development and Content

The survey asked about a wide range of migraine prevention approaches, both pharmacological and non-pharmacological, that were recommended by international guidelines on migraine treatment or mentioned in evidence reviews [6,12,13,14,27,28]. Respondents were asked: Which of the following supplements have you used to prevent migraine attacks? The supplements listed included magnesium, riboflavin, coenzyme Q10, feverfew and butterbur, with a free text option to note any other supplements. Where respondents reported use of Migradol (a commercial combination of magnesium and riboflavin), this was coded under both supplements. Respondents were also asked about use of complementary therapies for migraine prevention, including acupuncture, meditation or mindfulness practice, yoga or Tai Chi, biofeedback, massage, neurostimulation devices, with a free-text option to note other modalities. The question was: Which of the following non-medication approaches have you used to prevent migraine attacks? Response options to the questions on use of these treatments were: currently use; previously used—stopped because of side effects; previously used—stopped because it didn’t work; previously used—stopped for another reason; never used—would like to try; never used—don’t want to try. For another perspective on complementary therapy use, respondents were also asked whether they had seen a range of allied health professionals for migraine, including a chiropractor, osteopath, nutritionist or dietician, physiotherapist, occupational therapist, acupuncturist, naturopath or massage therapist. Response options were: seen in the last 12 months; seen in the past but >12 months ago; never seen—would like to; never seen—don’t want to.

Questions on sociodemographic measures (age, gender, ethnicity) were modelled on those used by Statistics NZ and the Ministry of Health. Respondents were asked: On average, on how many days a month do you have a headache? (If a headache lasted more than 1 day, count each day). We grouped respondents into those reporting 15 or more headache days per month and those reporting headache fewer than 15 days per month. The questionnaire was piloted on six individuals, most of whom had lived experience of migraine, with minor modifications made to survey design.

### 2.3. Data Analysis

Survey responses were exported into Microsoft Excel (version 2505) for cleaning, collation, and analysis. Demographic and clinical data were re-classified as necessary (e.g., age bands, current use of supplement or complementary therapy). Only descriptive and unweighted statistics, including response frequencies and percentages, were computed given the self-selecting nature of the sample, with missing responses removed from the analysis, assuming respondents who did not complete every question were similar to those who did. Chi-square tests were performed to test for differences in supplement and complementary therapy use between the high- and lower-frequency headache groups, using a significant level of 0.05.

## 3. Results

### 3.1. Survey Respondents

Survey respondents were predominantly female and NZ European/Other (Table 1). The demographic profile of respondents with headache on 15 days or more a month was similar to those with less frequent headache. Further characterization of the sample can be found elsewhere [3,11,29].

### 3.2. Supplement Use

Almost half (49%) of respondents were currently taking a supplement, and 40% had previously used one (Table 2). People with headache on 15 or more days a month were significantly more likely to be currently using a supplement than those with less frequent headache. Only 2% of people with high-frequency headache had never used a supplement for migraine, compared with 13% of people with less frequent headache.

**Figure 1:**
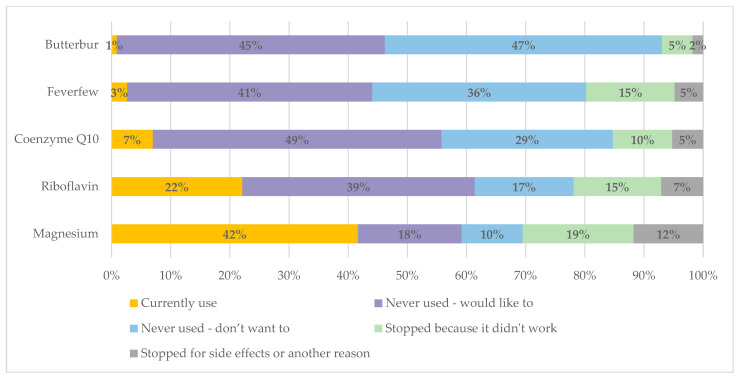
Use of specific supplements for migraine prevention.

The supplements most commonly used by survey respondents were magnesium and riboflavin (Figure 1). These were also the supplements most commonly used in the past but stopped for some reason (31% had previously used magnesium, 22% had previously used riboflavin). That left a large proportion of people who had never tried these supplements but would like to (18% for magnesium, 39% for riboflavin).

Few people were currently using coenzyme Q10 (7%), feverfew (3%) or butterbur (1%), but a higher proportion had used them in the past but stopped (15% had previously used coenzyme Q10; 20% had previously used feverfew; 7% had previously used butterbur) (Figure 1). Close to half of respondents had not used but would like to try one or more of these supplements.

For all supplements, the most common reason for discontinuation was because they did not work. Stopping because of side effects was much less frequent.

Survey respondents also mentioned other supplements they were using, including omega 3, vitamin D3, iron, vitamin C, lysine and multivitamins. Of people currently taking any supplements, just over half were taking one, 38% were taking two and 11% were taking between three and five supplements.

### 3.3. Complementary Therapy Use

More than half of respondents were currently using one or more complementary approaches, and only 15% had never tried any complementary therapy (Table 2). Fewer people with headache on 15 or more days a month had never tried any complementary therapy (5%) compared to those with less frequent headache (18%).

**Figure 2:**
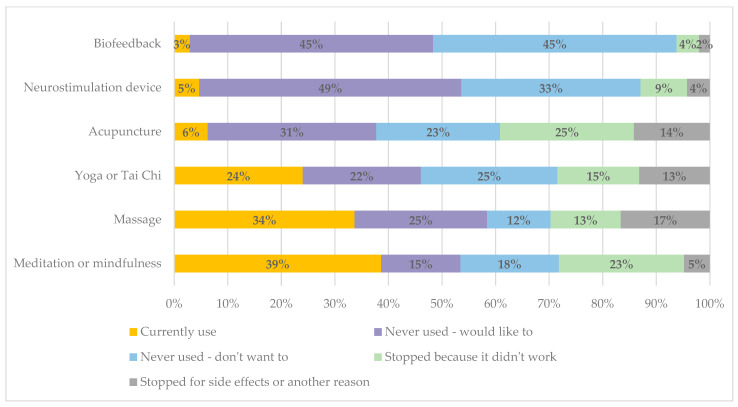
Use of specific complementary therapies for migraine prevention.

The most frequently used complementary therapies were meditation or mindfulness and massage, followed by yoga or Tai Chi (Figure 2). Few people were currently using the other approaches. Around a quarter of respondents had tried and stopped using meditation/mindfulness or acupuncture because it did not work. Stopping because of side effects was rare and more people stopped for ‘another reason’. Although we did not ask people to specify these reasons, in other survey questions, cost was mentioned as a barrier to using complementary therapies.

**Figure 3:**
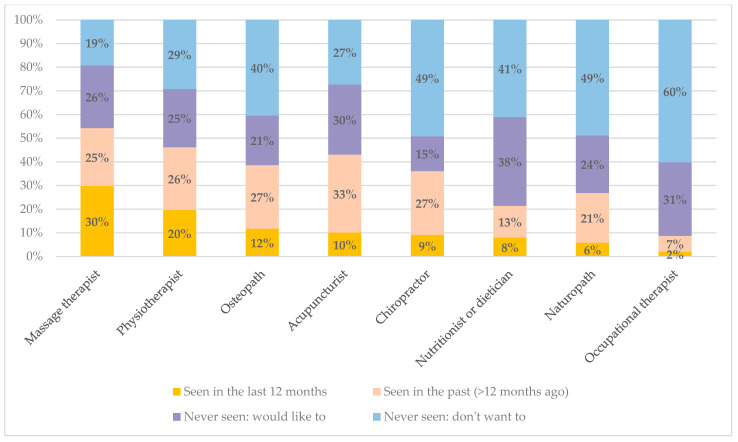
Allied health professionals seen for migraine.

In line with the reported current use of massage (which could include self-massage and massage delivered by family or friends), the allied health professional visited most in the last year was a massage therapist, by 30% of respondents (Figure 3). One in five had seen a physiotherapist in the last 12 months but other therapists had been recently seen by only a small proportion of respondents. Between 15 and 38% of respondents had not used these services before but would like to.

### 3.4. Combined Use of Preventive Treatments

Of those respondents who were currently taking a supplement, 55% were also currently taking a prescribed preventive medication, and 70% were currently using a complementary therapy. Of those respondents currently using complementary therapy, more than half (54%) were also taking a prescribed preventive medication. From all respondents who answered these questions (n = 485), one in five (20%) were currently using prescription medication, supplements, and complementary approaches for migraine prevention.

## 4. Discussion

In line with other research [8,23], this survey of people with migraine in NZ found that around half or more were currently using non-pharmacological treatments for migraine prevention. From a previous analysis, half of the survey respondents were taking at least one prescribed medication for migraine prevention [11]; hence, non-pharmacological approaches for migraine prevention were at least as common as prescribed medication. People with headache on 15 days or more a month were more likely to be using supplements and complementary therapies than those with less frequent headache, which demonstrates the drive and willingness to try different options among those with more severe disease [11].

The commonly used supplements among respondents were the ones with the best evidence and safety profiles (magnesium and riboflavin). However, around a quarter of those who had tried magnesium had stopped because it did not work, and a third of those who had tried riboflavin had stopped because it did not work, with smaller proportions stopping because of side effects. We were unable to assess whether respondents had stopped supplements because of inadequate dosage or treatment duration; this would be useful to explore in future research. Up to half of respondents reported wanting to try one of the supplements listed in the survey—likely barriers to use include cost, accessibility (not all pharmacies stock supplements at the doses specific for migraine treatment), and lack of information about these options.

For complementary therapies, the therapies currently used are those that are most accessible, such as meditation and mindfulness, which can be practiced alone and at home. Such self-management approaches can have a positive impact on pain severity and disability [30]. These therapies may also serve multiple purposes, not solely migraine prevention, and rates of current use may be overestimated, for example, if someone reported using meditation but this was primarily to manage anxiety rather than migraine. However, the commonly used therapies also had significant failure rates, with people having tried and stopped because of lack of efficacy or other reasons [31].

Few people were currently using neurostimulation devices or biofeedback or had tried these in the past, but nearly half of respondents would like to try these, indicating a large unmet need for such treatments, both of which have a strong evidence base for effectiveness [13,18]. Apart from massage therapists and physiotherapists, visits to complementary therapy services in the past year were uncommon. From previous analyses, cost, waiting times, and inability to obtain appointments were reported as the main reasons for being unable to see health professionals, including complementary therapists [29]. Although not all respondents wanted to try complementary therapies, between 15 and 38% had never tried but would like to try one or another of these therapies, again signaling a level of unmet need for additional effective treatments.

The role of medical professionals in discussing and facilitating access to non-pharmacological migraine prevention is not well described. From a survey of attendees at a headache clinic in the UK, the commonest source of information about complementary therapies was friends or relatives [22], and many people with migraine using complementary therapies do not discuss this with their healthcare provider [22,23]. Although international guidelines on migraine treatment include discussion of non-pharmacological approaches [6,28], this may be out of scope for primary care clinicians who have limited time in short appointments to address options for migraine prevention beyond medication. In NZ, increased use of health coaches, health improvement practitioners and other allied health professionals has been recognized as a way to expand and improve treatment options and outcomes for people with migraine [32]. However, many complementary therapies, such as acupuncture, massage therapy, biofeedback and physiotherapy, are fee-for-service and may require multiple visits in order to experience a significant effect. Without additional support or funding for these services, they will not be an affordable treatment option for many. Given the current pressures on the health system, with long waiting times to see primary and secondary care providers [33], more research into the cost-effectiveness of non-medication approaches to migraine prevention could help inform whether subsidies for evidence-based complementary therapies would be beneficial for both patients and healthcare providers.

In this survey, we did not specifically ask about lifestyle measures that all people with migraine, and all people in general, are advised to undertake, such as regular exercise, a healthy diet and sufficient sleep. These are sometimes listed among non-pharmacological treatments [8,24], highlighting the lack of a clear definition of what constitutes non-pharmacological treatment for migraine prevention. For future research, we recommend asking about a comprehensive list of non-pharmacological approaches and reporting separately on supplements, complementary therapies, lifestyle factors and approaches with the highest level of evidence.

This was a cross-sectional survey with some limitations. Respondents were asked about headache frequency over the last month to minimize recall bias from asking about a longer timeframe, in the context of an online population survey where most respondents would not be completing daily headache diaries. This means respondents who reported 15 or more headache days a month did not meet the classification criteria for chronic migraine, so results for this group may not be exactly comparable to those with 15 or more headache days for at least three months. However, there may be little difference between these groups, given that headache frequency in migraine is known to fluctuate over time, with people crossing the definitions of episodic to chronic migraine and back over relatively short time periods [34,35].

The self-selecting nature of the sample, recruitment methods and online survey delivery mean that results are not generalizable to all people with migraine in NZ, and likely explains why the sample peak of 45–54 years is a little older than other surveys, where migraine prevalence peaks around 40–44 years [36]. Around 22% of the sample reported headache on 15 days or more a month, which is more than expected, given that international studies find around 5–10% of people with migraine have chronic migraine [5,37,38]. This suggests that survey respondents had a higher level of need for migraine treatment than would be found in a general migraine population survey and may be more likely to try supplements and complementary therapies. Therefore, the survey results may overestimate the proportion of people who would like to try different therapies or consult different allied health professionals. People with less access to digital resources or less engagement with social media, patient organizations, and health networks would also be less likely to complete the survey, which includes Indigenous Māori and Pacific peoples, who were under-represented. More research into the management of migraine in different ethnic groups is needed in NZ and may be achievable with the inclusion of questions about migraine in the nationally representative NZ Health Survey in 2023/24 [39].

From a sample of people with migraine, including many experiencing a high frequency of headache, this survey reveals a high level of unmet need for effective migraine prevention in NZ, both pharmacologically and non-pharmacologically. At the time of the survey, access to the new migraine preventive medications that target calcitonin gene-related peptide (CGRP) was very limited, with only two monoclonal antibodies (erenumab and galcanezumab) available, but only through private purchase. As of May 2025, none of the anti-CGRP migraine medications were funded in NZ. Improved access to these new migraine-specific preventive medications could address a large part of this unmet need, as they are now recommended as a first-line treatment due to a safety, efficacy, and tolerability profile that is superior to existing treatments [40,41,42].

This survey also confirmed that there is no approach to migraine prevention that is universally effective. Much more research is needed to understand the underlying pathophysiology of migraine disease and identify alternative treatments for those who continue to have frequent and severe migraine attacks despite trying many current therapies.

## Figures and Tables

**Table 1:** Characteristics of the survey respondents by headache frequency.

Characteristic	Headache 15+ Days/Month (n/col%)	Headache <15 Days/Month (n/col%)	Total (n/col%)
Age group			
<18 years	1 (1%)	1 (<1%)	2 (<1%)
18−24 years	5 (4%)	15 (4%)	20 (4%)
25−34 years	16 (14%)	64 (16%)	80 (15%)
35−44 years	30 (25%)	93 (23%)	123 (23%)
45−54 years	35 (30%)	120 (29%)	155 (29%)
55−64 years	15 (13%)	55 (13%)	70 (13%)
65+ years	7 (6%)	24 (6%)	31 (6%)
Missing data	9 (8%)	40 (10%)	49 (9%)
Gender			
Female	96 (81%)	337 (82%)	433 (82%)
Male	10 (9%)	31 (8%)	41 (8%)
Another gender	3 (3%)	5 (1%)	8 2%)
Missing data	9 (8%)	39 (10%)	48 (9%)
Prioritized ethnicity *			
Māori	7 (6%)	32 (8%)	39 (7%)
Pacific	0 (0%)	6 (2%)	6 (1%)
Asian	2 (2%)	21 (5%)	23 (4%)
NZ European/Other	99 (84%)	310 (75%)	409 (77%)
Missing data	10 (9%)	43 (10%)	53 (10%)
Total	118	412	530

* For multiple ethnicity responses, ethnicity was prioritized in order of Māori, Pacific, Asian, European/Other.

**Table 2:** Use of supplements and complementary therapies in survey respondents by headache frequency.

	Headache 15+ Days/Month (n/col%)	Headache <15 Days/Month (n/col%)	Total (n/col%)	Chi-Square
Use of supplements ^1^				
Current use	62 (56%)	178 (47%)	240 (49%)	
Previous use	47 (42%)	150 (40%)	197 (40%)	
Never used	2 (2%)	49 (13%)	51 (11%)	11.725, *p* = 0.003
Use of complementary therapies ^2^				
Current use	73 (66%)	212 (56%)	285 (58%)	
Previous use	32 (29%)	101 (27%)	133 (27%)	
Never used	6 (5%)	67 (18%)	73 (15%)	10.27, *p* = 0.006

^1^ 42 missing responses (7 15+ headache days/month, 35 headache <15 days/month); ^2^ 39 missing responses (7 15+ headache days/month, 32 <15 headache days/month).

## Data Availability

Data are not publicly available. Study participants were not asked for permission, and nor was ethical approval granted for data to be shared publicly.

## Abbreviations

The following abbreviations are used in this manuscript.

CGRP	Calcitonin gene-related peptide
MFANZ	Migraine Foundation Aotearoa New Zealand
NZ	Aotearoa New Zealand

## References

[1] Steiner T.J., Husøy A., Stovner L.J. (2024). GBD2021: Headache Disorders and Global Lost Health—A Focus on Children, and a View Forward. J. Headache Pain..

[2] Global Burden of Disease Collaborative Network (2024). Global Burden of Disease Study 2021 (GBD 2021) Results. https://vizhub.healthdata.org/gbd-results/.

[3] Garrett S.M., Imlach F. (2024). The Impact of Living with Migraine Disease in Aotearoa New Zealand. N. Z. Med. J..

[4] Headache Classification Committee of the International Headache Society (IHS) (2018). The International Classification of Headache Disorders, 3rd Edition. Cephalalgia.

[5] Lipton R.B., Manack Adams A., Buse D.C., Fanning K.M., Reed M.L. (2016). A Comparison of the Chronic Migraine Epidemiology and Outcomes (CaMEO) Study and American Migraine Prevalence and Prevention (AMPP) Study: Demographics and Headache-Related Disability. Headache.

[6] Ailani J., Burch R.C., Robbins M.S. (2021). The American Headache Society Consensus Statement: Update on Integrating New Migraine Treatments into Clinical Practice. Headache J. Head Face Pain.

[7] Manack Adams A., Lanteri-Minet M., Leroux E., Katsarava Z., Lipton R.B., Sakai F., Matharu M., Fanning K., Sommer K., Seminerio M. (2024). Characterizing Barriers to Care in Migraine: Multicountry Results from the Chronic Migraine Epidemiology and Outcomes-International (CaMEO-I) Study. J. Headache Pain.

[8] Groth M., Katsarava Z., Ehrlich M. (2022). Results of the GErman Migraine PatIent Survey on Medical Care and PrOPhylactic Treatment Experience (EPISCOPE). Sci. Rep..

[9] Hepp Z., Bloudek L.M., Varon S.F. (2014). Systematic Review of Migraine Prophylaxis Adherence and Persistence. J. Manag. Care Pharm..

[10] Hepp Z., Dodick D.W., Varon S.F., Chia J., Matthew N., Gillard P., Hansen R.N., Devine E.B. (2017). Persistence and Switching Patterns of Oral Migraine Prophylactic Medications among Patients with Chronic Migraine: A Retrospective Claims Analysis. Cephalalgia.

[11] Imlach F., Garrett S. (2024). Use of Medications for Migraine in Aotearoa New Zealand. N. Z. Med. J..

[12] Wells R.E., Beuthin J., Granetzke L. (2019). Complementary and Integrative Medicine for Episodic Migraine: An Update of Evidence from the Last 3 Years. Curr. Pain Headache Rep..

[13] Patel P., Minen M.T. (2019). Complementary and Integrative Health Treatments for Migraine. J. Neuroophthalmol..

[14] Kuruvilla D., Erwin Wells R. (2019). Evidence-Based Integrative Treatments for Headache. Headache J. Head Face Pain.

[15] Millstine D., Chen C.Y., Bauer B. (2017). Complementary and Integrative Medicine in the Management of Headache. BMJ.

[16] Karakurum Göksel B. (2012). The Use of Complementary and Alternative Medicine in Patients with Migraine. Arch. Neuropsychiatry.

[17] Posadzki P., Albedah A.M., Khalil M.M., Alqaed M.S., Lee M.S., Ernst E., Car J. (2015). Complementary and Alternative Medicine for the Prevention and Treatment of Migraine Headache: An Overview of Systematic Reviews. Focus Altern. Complement. Ther..

[18] Han X., Yu S. (2023). Non-Pharmacological Treatment for Chronic Migraine. Curr. Pain. Headache Rep..

[19] Moisset X., Pereira B., Ciampi De Andrade D., Fontaine D., Lantéri-Minet M., Mawet J. (2020). Neuromodulation Techniques for Acute and Preventive Migraine Treatment: A Systematic Review and Meta-Analysis of Randomized Controlled Trials. J. Headache Pain.

[20] Gaul C., Eismann R., Schmidt T., May A., Leinisch E., Wieser T., Evers S., Henkel K., Franz G., Zierz S. (2009). Use of Complementary and Alternative Medicine in Patients Suffering from Primary Headache Disorders. Cephalalgia.

[21] Rossi P., Di Lorenzo G., Malpezzi M.G., Faroni J., Cesarino F., Di Lorenzo C., Nappi G. (2005). Prevalence, Pattern and Predictors of Use of Complementary and Alternative Medicine (CAM) in Migraine Patients Attending a Headache Clinic in Italy. Cephalalgia.

[22] Lambert T.D., Morrison K.E., Edwards J., Clarke C.E. (2010). The Use of Complementary and Alternative Medicine by Patients Attending a UK Headache Clinic. Complement. Ther. Med..

[23] Wells R.E., Bertisch S.M., Buettner C., Phillips R.S., Mccarthy E.P., Bertisch S.M., Buettner C., Phillips R.S., Mccarthy E.P. (2011). Complementary and Alternative Medicine Use Among Adults With Migraines/Severe Headaches. Headache J. Head Face Pain.

[24] Marupuru S., Almatruk Z., Slack M.K., Axon D.R. (2023). Use of Pharmacological and Non-Pharmacological Strategies by Community-Dwelling Adults to Manage Migraine: A Systematic Review. Clin. Pract..

[25] Lipton R.B., Dodick D., Sadovsky R.E.A.A., Kolodner K., Endicott J., Hettiarachchi J., Harrison W. (2003). A Self-Administered Screener for Migraine in Primary Care The ID Migraine^TM^ Validation Study. Neurology.

[26] Cousins G., Hijazze S., Van De Laar F.A., Fahey T. (2011). Diagnostic Accuracy of the ID Migraine: A Systematic Review and Meta-Analysis. Headache J. Head Face Pain.

[27] British Association for the Study of Headache (BASH) (2019). National Headache Management System for Adults 2019.

[28] Diener H.-C., Holle-Lee D., Nägel S., Dresler T., Gaul C., Gö Bel H., Heinze-Kuhn K., Jü Rgens T., Kropp P., Meyer B. (2019). Treatment of Migraine Attacks and Prevention of Migraine: Guidelines by the German Migraine and Headache Society and the German Society of Neurology. Clin. Transl. Neurosci..

[29] McInnernay B., Imlach F., Kennedy J., Garrett S. (2024). Evaluating Barriers for Effective Migraine Management in Aotearoa New Zealand. J. Prim. Health Care.

[30] Probyn K., Bowers H., Mistry D., Caldwell F., Underwood M., Patel S., Sandhu H.K., Matharu M., Pincus T. (2017). Non-Pharmacological Self-Management for People Living with Migraine or Tension-Type Headache: A Systematic Review Including Analysis of Intervention Components. BMJ Open.

[31] Tepper S.J., Cirillo J., Kim E., L’Italien G., Tweedie J.M., Lodaya K., Riley D., Pathan F., Antaki N., Nathanson B.H. (2023). The Temporal Trend of Placebo Response in Migraine Prevention from 1990 to 2021: A Systematic Literature Review and Meta-Analysis with Regression. J. Headache Pain.

[32] Sanderson J., Imlach F., Kennedy J., Garrett S. (2024). Primary Care Clinicians’ Perspectives on Migraine Management in Aotearoa New Zealand: A Qualitative Study. J. Prim. Health Care.

[33] IPSOS Global Health Service Monitor (2024). IPSOS Health Service Report 2024. New Zealand Edition.

[34] Dumkrieger G.M., Ishii R., Goadsby P.J. (2025). Flexible Modeling of Headache Frequency Fluctuations in Migraine with Hidden Markov Models. Headache J. Head Face Pain.

[35] Serrano D., Lipton R.B., Scher A.I., Reed M.L., Stewart W.F., Manack Adams A., Buse D.C. (2017). Fluctuations in Episodic and Chronic Migraine Status over the Course of 1 Year: Implications for Diagnosis, Treatment and Clinical Trial Design. J. Headache Pain..

[36] Dong L., Dong W., Jin Y., Jiang Y., Li Z., Yu D. (2024). The Global Burden of Migraine: A 30-Year Trend Review and Future Projections by Age, Sex, Country, and Region. Pain Ther..

[37] May A., Schulte L.H. (2016). Chronic Migraine: Risk Factors, Mechanisms and Treatment. Nat. Rev. Neurol..

[38] Adams A.M., Buse D.C., Leroux E., Lanteri-Minet M., Sakai F., Matharu M.S., Katsarava Z., Reed M.L., Fanning K., Sommer K. (2023). Chronic Migraine Epidemiology and Outcomes—International (CaMEO-I) Study: Methods and Multi-Country Baseline Findings for Diagnosis Rates and Care. Cephalalgia.

[39] Migraine Foundation Aotearoa New Zealand (2023). New Migraine Questions in the Next New Zealand Health Survey.

[40] Kubota G.T. (2022). It Is Time Anti-CGRP Monoclonal Antibodies Be Considered First-Line Prophylaxis for Migraine. Arq. Neuropsiquiatr..

[41] Sacco S., Amin F.M., Ashina M., Bendtsen L., Deligianni C.I., Gil-Gouveia R., Katsarava Z., MaassenVanDenBrink A., Martelletti P., Mitsikostas D.-D. (2022). European Headache Federation Guideline on the Use of Monoclonal Antibodies Targeting the Calcitonin Gene Related Peptide Pathway for Migraine Prevention—2022 Update. J. Headache Pain.

[42] Charles A.C., Digre K.B., Goadsby P.J., Robbins M.S., Hershey A. (2024). Calcitonin Gene-Related Peptide-Targeting Therapies Are a First-Line Option for the Prevention of Migraine: An American Headache Society Position Statement Update. Headache J. Head Face Pain.

